# Remission for Loss of Odontogenic Potential in a New Micromilieu *In Vitro*

**DOI:** 10.1371/journal.pone.0152893

**Published:** 2016-04-06

**Authors:** Yunfei Zheng, Jinglei Cai, Andrew Paul Hutchins, Lingfei Jia, Pengfei Liu, Dandan Yang, Shubin Chen, Lihong Ge, Duanqing Pei, Shicheng Wei

**Affiliations:** 1 Department of Oral and Maxillofacial Surgery, Peking University School and Hospital of Stomatology, Beijing 100081, P.R. China; 2 Institute for Stem Cell Biology and Regenerative Medicine, Guangzhou Institute of Biomedicine and Health, Chinese Academy of Sciences, Guangzhou 510530, P.R. China; 3 Central Laboratory, Peking University School and Hospital of Stomatology, Beijing 100081, P.R. China; 4 Department of Pediatric Dentistry, Peking University School and Hospital of Stomatology, Beijing 100081, P.R. China; Laboratoire de Biologie du Développement de Villefranche-sur-Mer, FRANCE

## Abstract

During embryonic organogenesis, the odontogenic potential resides in dental mesenchyme from the bud stage until birth. Mouse dental mesenchymal cells (mDMCs) isolated from the inductive dental mesenchyme of developing molars are frequently used in the context of tooth development and regeneration. We wondered if and how the odontogenic potential could be retained when mDMCs were cultured *in vitro*. In the present study, we undertook to test the odontogenic potential of cultured mDMCs and attempted to maintain the potential during culturing. We found that cultured mDMCs could retain the odontogenic potential for 24 h with a ratio of 60% for tooth formation, but mDMCs were incapable of supporting tooth formation after more than 24 h in culture. This loss of odontogenic potential was accompanied by widespread transcriptomic alteration and, specifically, the downregulation of some dental mesenchyme-specific genes, such as *Pax9*, *Msx1*, and *Pdgfrα*. To prolong the odontogenic potential of mDMCs *in vitro*, we then cultured mDMCs in a serum-free medium with Knockout Serum Replacement (KSR) and growth factors (fibroblastic growth factor 2 and epidermal growth factor). In this new micromilieu, mDMCs could maintain the odontogenic potential for 48 h with tooth formation ratio of 50%. Moreover, mDMCs cultured in KSR-supplemented medium gave rise to tooth-like structures when recombined with non-dental second-arch epithelium. Among the supplements, KSR is essential for the survival and adhesion of mDMCs, and both Egf and Fgf2 induced the expression of certain dental mesenchyme-related genes. Taken together, our results demonstrated that the transcriptomic changes responded to the alteration of odontogenic potential in cultured mDMCs and a new micromilieu partly retained this potential *in vitro*, providing insight into the long-term maintenance of odontogenic potential in mDMCs.

## Introduction

Vertebrate organs, including tooth, develop upon interactions typically between epithelial and mesenchymal tissues, with one tissue component producing inductive stimuli and another one responding to the induction [1–2]. Odontogenic potential represents an instructive induction capability of a tissue to induce gene expression in an adjacent tissue and to initiate tooth development [3]. In mice, the odontogenic potential shifts from the epithelial compartment to dental mesenchyme at the early bud stage [4–5]. The inductive dental mesenchyme is able to determine the odontogenic fate of dental and non-dental epithelium [4, 6–9].

Dental mesenchymal cells isolated from prenatal or postnatal tooth germs participate in whole-tooth regeneration in mice, pigs, and rats [10–12]. Mouse dental mesenchymal cells (mDMCs) give rise to the whole dental pulp mesenchyme, including the odontoblasts. However, preparation of embryonic cells is time-consuming and acquisition of embryos at the right stage is laborious. In addition, embryonic tooth germ cells are inaccessible in the adult, and xenogenic embryonic tooth germ cells suffer from immune rejection, especially in humans. Thus, several easily available cell sources potentially could be employed to regenerate a whole tooth, including ecto-mesenchymal cells prepared from postnatal teeth, immortalized cell lines, and induced pluripotent stem cells.

Postnatal dental pulp contains stem cells that are capable of generating a dentin-like structure lined with odontoblast-like cells [13]. Although dental pulp stem cells potentially could be used for dentinal repair of teeth [14], they do not induce tooth formation when recombined with dental epithelium [3]. Several immortalized cell lines have been established from mouse and human dental mesenchymal cells and display similar characteristics of the primary cells [15–19]. These immortalized cells express tooth-specific genes and can differentiate towards a odontoblast fate. But immortalized cell lines from dental mesenchyme of the bell stage fail to induce the morphogenesis of tooth [20]. Recently, mouse induced pluripotent stem cells showed the potential to differentiate into mDMC-like cells through neural crest-like cells (NCLCs) [21]. Although the recombinant with mDMC-like cells and incisor dental epithelium demonstrated calcified tooth germ-like structures with bone [22], whether the mDMC-like cells possess odontogenic potential was not identified. Collectively, a cell source with odontogenic potential other than mDMCs has not been reported. Efficient strategies for the culture of odontogenic mDMCs are essential for the study of tooth development and would provide opportunities in regenerative medicine.

However, *in vitro* expansion of mDMCs without impairing the odontogenic potential remains a great challenge. The odontogenic potential of mDMCs of embryonic day 14 is lost in the course of culturing [20]. Similarly, the loss of potential has also been reported for hematopoietic stem cells (HSCs). The potential of HSCs expanded *in vitro* is impaired in subsequent *in vivo* regenerative assays [23]. Various cytokine cocktails have been used to support HSC growth *in vitro*, and many factors are found to promote the survival and regenerative potential of HSCs [24–25]. Thus, we wonder if supplementation of growth factors would facilitate the maintenance of odontogenic potential *in vitro*.

**Fig 1:**
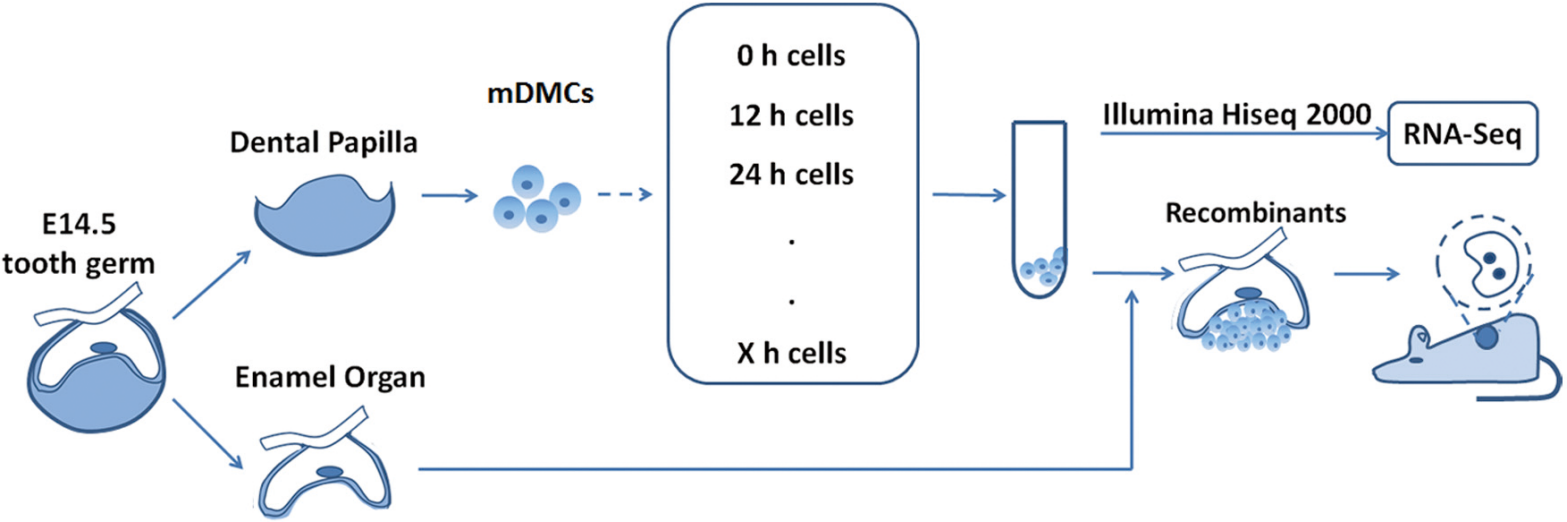
Design of the study. Dental mesenchyme tissues from embryonic day 14.5 (E14.5) mice were digested with trypsin. Freshly isolated mouse dental mesenchymal cells (mDMCs) were divided into three groups: One group was recombined with embryonic dental epithelial and cultured in kidney; the second was submitted for RNA-seq; the third was cultured *in vitro* and harvested at indicated time points.

In the present study, we have examined the odontogenic potential of cultured mDMCs and found a new approach to maintain the potential during culture based on the transcriptomic data of mDMCs (Fig 1). Our results showed that cultured mDMCs rapidly lost odontogenic potential. RNA-seq analysis revealed a rapid loss of the dental mesenchymal signature in cultured mDMCs and a deviation away from the neural crest. To avoid cell apoptosis and cell differentiation towards fibroblasts, Knockout Serum Replacement (KSR) was used to culture mDMCs instead of fetal bovine serum (FBS). Fibroblastic growth factor 2 (Fgf2), and epidermal growth factor (Egf) that are essential for the development of neural crest and tooth were also used. The new culture micromilieu with KSR/Fgf2/Egf retained the expression of some dental mesenchyme-specific genes and delayed the loss of odontogenic potential by 24 h. Our work revealed the characteristics and behavior of mDMCs in culture and suggested routes for tooth regeneration from cultured mDMCs.

## Materials and Methods

### Cell culture

All animal procedures were approved by the Animal Care Committee of Peking University and Guangzhou Institutes of Biomedicine and Health Ethical Committee (permit Number: CMU-B20100106). Tooth germs of the mandibular first molar in embryonic day 14.5 (E14.5) mouse embryos were dissected using fine needles and treated with dispase to separate dental mesenchyme from the epithelium. The isolated dental mesenchyme was digested with trypsin and filtered through a 40-μm cell sieve to obtain single cells. mDMCs were cultured at a density of 1 × 10^4^/cm^2^ in Dulbecco’s modified Eagle’s medium (DMEM; Gibco, Grand Island, NY) supplemented with 10% FBS (Gibco), 100 U/ml penicillin, and 100 g/ml streptomycin. To prolong the odontogenic potential of mDMCs, freshly isolated cells were cultured on gelatin-coated plates in a new medium with 10% KSR (Invitrogen, Carlsbad, CA), 20 ng/ml FGF2 (R&D system) and 20 ng/ml EGF (R&D system).

### Tissue recombination and subrenal culture

Freshly isolated and cultured mDMCs were harvested at indicated time points. About 1 × 10^5^ mDMCs were spun down to make a cell pellet and left in the centrifuge tube for aggregation for 2 h in DMEM + 10%FBS. The cell pellets was then recombined with freshly isolated E14.5 dental epithelium as previously described [9]. All recombinants were further cultured for 24 h prior to subrenal culture in adult male ICR mice. The host mice were sacrificed 3 weeks later and the grafted tissues were harvested. Grafts were fixed in 4% PFA/PBS, embedded in paraffin, and sectioned at 7 μm. Sections were stained with H&E for histological analysis.

### RNA isolation and sequencing

Total RNA from freshly isolated and cultured mDMCs was extracted using the RNeasy mini kit and RNase-Free DNase set (Qiagen GmbH, Hilden, Germany). RNA library from each sample was prepared according to the instructions with the Illumina TruSeq RNA kit and sequenced on an Illumina Hiseq 2000 in duplicate or triplicate. Raw data of the performed RNA-Seq experiments have been recorded in the GEO public database (accession number: GSE78228).

### Quantitative reverse transcription PCR (qRT-PCR)

Total RNA was extracted with Trizol and complementary DNA was synthesized using an RT-PCR kit (TaKaRa Bio, Otsu, Japan). Real-time PCR was performed in triplicates in a Thermal CyclerDice7^™^ RealTime System with SYBR Green Premix EXTaq^™^ (Takara Bio). The primers are listed in Table 1. RNA expression was normalized to Actin and freshly isolated samples using the 2^-ΔΔCt^ method.

**Table 1:** Primers for quantitative real-time PCR.

	Forward	Reverse
**Actin**	5’-GGC TGT ATT CCC CTC CAT CG-3’	5’-CCA GTT GGT AAC AAT GCC ATG T-3’
**Dlx1**	5’-GGC TAC CCC TAC GTC AAC TC-3’	5’-TTT TTC CCT TTG CCG TTA AAG C-3’
**Lhx6**	5’-CAT TGA GAG TCA GGT ACA GTG C-3’	5’-GGG CCG TCC AAA TCA GCT T-3’
**Pax9**	5’- CAT TCG GCT TCG CAT CGT G -3’	5’- CTC CCG GCA AAA TCG AAC C-3’
**Bmp4**	5’-TTC CTG GTA ACC GAA TGC TGA-3’	5’-CCT GAA TCT CGG CGA CTT TTT-3’
**Dlx2**	5'- CTA CGG CAC CAG TTC GTC TC -3'	5'- CCG TTC ACT ATT CGG ATT TCA GG-3'
**Msx1**	5'- GCA CAA GAC CAA CCG CAAG-3'	5'- CGC TCG GCA ATA GAC AGG T-3'
**Fgf10**	5'- GCA GGC AAA TGT ATG TGG CAT-3'	5'- ATG TTT GGA TCG TCA TGG GGA-3'
**Alp**	5’- CCA ACT CTT TTG TGC CAG AGA-3’	5'- GGC TAC ATT GGT GTT GAG CTT TT-3'
**Wnt5a**	5'- CAA CTG GCA GGA CTT TCT CAA-3'	5'- CAT CTC CGA TGC CGG AAC T-3'

### Immunofluorescence analysis

mDMCs were cultured for the indicated periods and fixed in 4% paraformaldehyde. Fixed cells were treated with 0.1% Triton X-100 for 5 min, blocked with 5% BSA for 60 min, and further incubated with primary antibodies at 4°C overnight. The primary antibodies were anti-Msx1 goat monoclonal IgG (1:100; Santa Cruz Biotechnology, Santa Cruz, CA), anti-Pax9 rabbit polyclonal IgG (1:100; Cell Signaling, Danvers, MA), anti-Pdgfrα rabbit monoclonal IgG (1:100; Origene, Rockville, MD), anti-p75 rabbit polyclonal IgG (1:50; Santa Cruz), and anti-Bmp4 rabbit polyclonal IgG (1:500; Abcam, UK). The secondary antibodies Alexa Fluor 568 donkey Anti-rabbit IgG (1:1000), FITC-conjugated goat anti-rabbit IgG (1:1000) and FITC-conjugated donkey anti-goat IgG (1:1000) were applied for 60 min. The cells were counterstained with DAPI (5 μg/mL) and sealed with mounting medium. Blocking buffer with BSA was used as a negative control.

### Cell proliferation analysis

Cell counting kit 8 (CCK8; Dojindo, Tokyo, Japan) was used to measure the cell viability according to the protocol. Briefly, freshly isolated mDMCs were seeded in a 96-well plate and cultured in the incubator for 6 h to allow cell attachment. Subsequently, the media was replaced with FBS- or KSR-supplemented medium. After indicated time, 10 μl CCK-8 solution was added to each well and incubated for 3 h at 37°C. The optical density was measured at an absorbance of 450 nm using a microplate reader (ELx800; BioTek Instruments, Inc., Winooski, VT, USA).

### Bioinformatics analysis

RNA-sequencing reads were trimmed for adaptor sequence, mapped to the mouse transcriptome (mm10, Ensembl v73) and then aligned using bowtie (v1.0.1) and RSEM (v1.2.12). Differentially-expressed genes were identified using DESeq2 (v1.12.0); a *p*-value <0.05 and fold change >2 were used as the threshold to define significant differences in gene expression. The Database for Annotation, Visualization and Integrated Discovery was used to determine the GO categories and KEGG pathways using the entire mouse transcriptome as background gene set. The appropriate modules in glbase were used for hierarchical clustering and principal components analysis (PCA) [26]. Other RNA-seq was reanalyzed from GSE39918 [27], GSE55966 [28] and GSE29278 [29].

### Statistical analysis

Statistical analyses were performed using the SPSS for Windows software package (v18; SPSS Inc., Chicago, IL). Data from at least three independent experiments were used for analysis. The data were shown as means ± standard deviations (SD) and differences among groups were analyzed using one-way ANOVA. A two-tailed *p* value <0.05 was considered to be statistically significant.

## Results

### Loss of odontogenic potential in mouse dental mesenchymal cells

**Fig 2:**
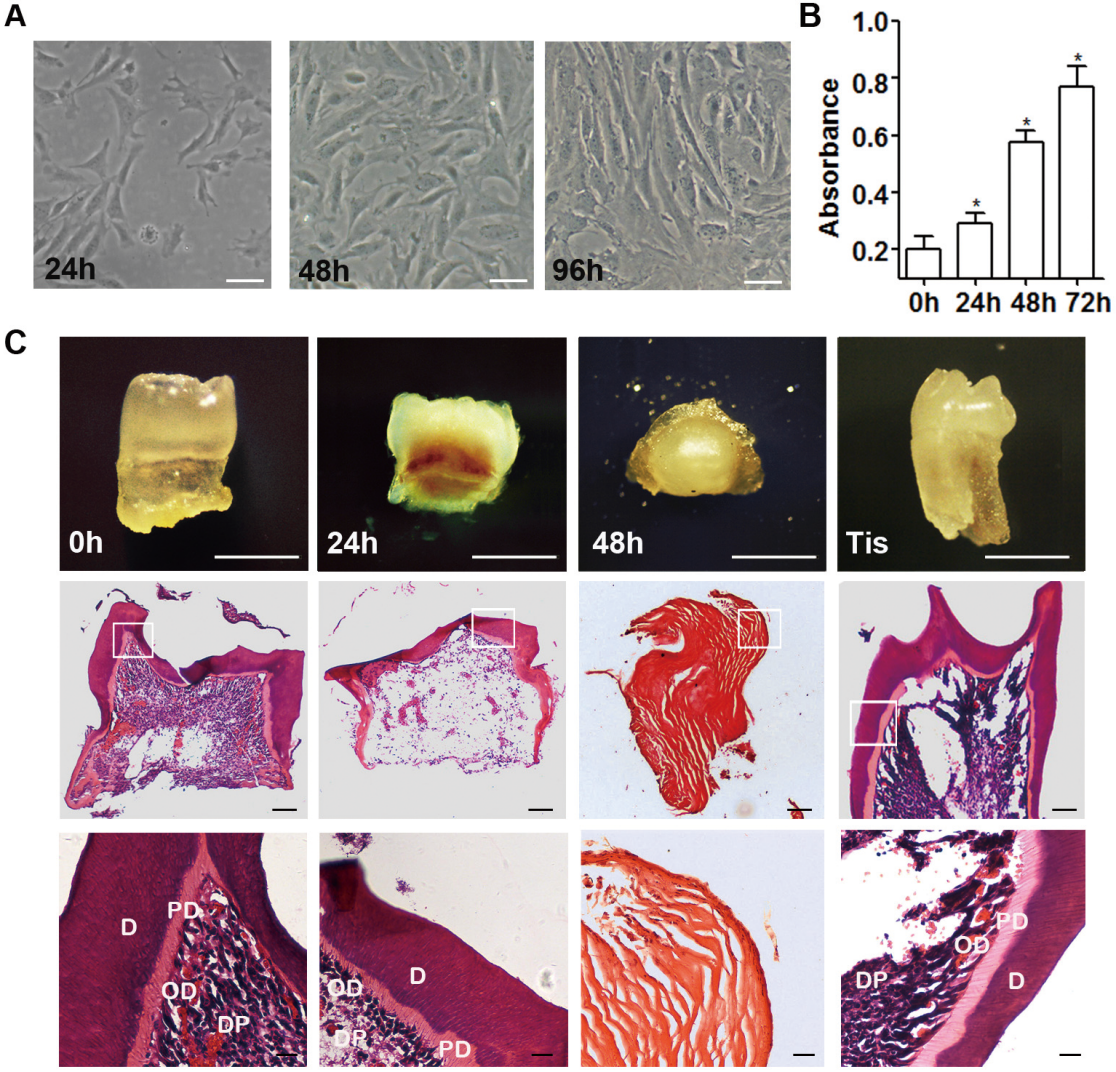
Impaired odontogenic potential in cultured mDMCs. **(A)** mDMCs possess a fibroblast-like or elliptic morphology. Cell density increased significantly and the morphological alteration was visible from 24 h to 96 h. **(B)** Cells continued to proliferate in culture and cell quantity doubled in 48 h. **(C)** Recombinants with freshly isolated mDMCs and cells cultured for 24 h formed tooth after subrenal culture, but recombinants with cells cultured for 48 h formed cysts. Recombinants with E14.5 dental mesenchyme (Tis) were used as positive control. H&E staining showed the presence of tooth structure or eosinophilic amorphous matrix. D, dentin; PD, predentin; DP, dental pulp; OD, odontoblast. Scale bar: A, B = 50 μm; C = 500 μm.

Among the isolated mDMCs, some displayed a spindle-shaped, fibroblast-like morphology and others an elliptic morphology when adhering to the plates (Fig 2A). The cells continued to proliferate in culture and cell quantity doubled in 48 h (Fig 2B). When recombined with E14.5 dental epithelium, both freshly isolated mDMCs and molar mesenchyme tissues developed into teeth with well-differentiated odontoblasts after 3 weeks of subrenal culture (Fig 2C). The tooth-formation ratio for freshly isolated cells was 21/28 and for molar mesenchyme tissue was 11/11. The first- (120 h) and second-passage (192 h) mDMCs showed no tooth formation when recombined with E14.5 dental epithelium, suggesting that the odontogenic potential was lost during *in vitro* culture (Table 2). Given the possible influence of culture duration, the odontogenic potential of cells cultured for shorter periods was then examined. Recombinants with cells cultured for 48 h failed to develop into dental tissue, forming cysts with an amorphous matrix. In contrast, recombinants with cells cultured for 24 h gave rise to well-organized, tooth-like structures with a ratio of 17/28 (Fig 2C, Table 2). Collectively, these data demonstrated the culture-induced impairment of odontogenic potential in mDMCs, and mDMCs lost their odontogenic potential after 24 h in culture.

**Table 2:** Ratios of tooth formation in tissue recombination assays.

*Type of recombinant*		*Culture medium*	*No*. *of recombinants*	*No*. *of tooth formation*	*Tooth formation ratio (%)*
*Mesenchyme*	*Epithelium*				
Dm E14.5	De E14.5	---	11	11	100
mDMCs 0h	De E14.5	M-FBS	28	21	78.8
mDMCs 24h	De E14.5	M-FBS	28	17	60.7
mDMCs 48h	De E14.5	M-FBS	30	0	0
mDMCs 120h	De E14.5	M-FBS	28	0	0
mDMCs 192h	De E14.5	M-FBS	32	0	0
mDMCs 0h	De E14.5	M-KSR	5	5	100
mDMCs 24h	De E14.5	M-KSR	27	14	51.9
mDMCs 48h	De E14.5	M-KSR	30	15	50
mDMCs 0h	Se E10.5	M-KSR	18	7	38.8
mDMCs 24h	Se E10.5	M-KSR	14	4	28.6
mDMCs 48h	Se E10.5	M-KSR	14	3	21.4

Abbreviations: Dm, dental mesenchyme; De, dental epithelium; Se, second-arch epithelium; mDMCs, mouse dental mesenchymal cells; M-FBS, FBS supplemented medium; M-KSR, KSR supplemented medium.

### Changes in the transcriptome profiles induced *in vitro*

**Fig 3:**
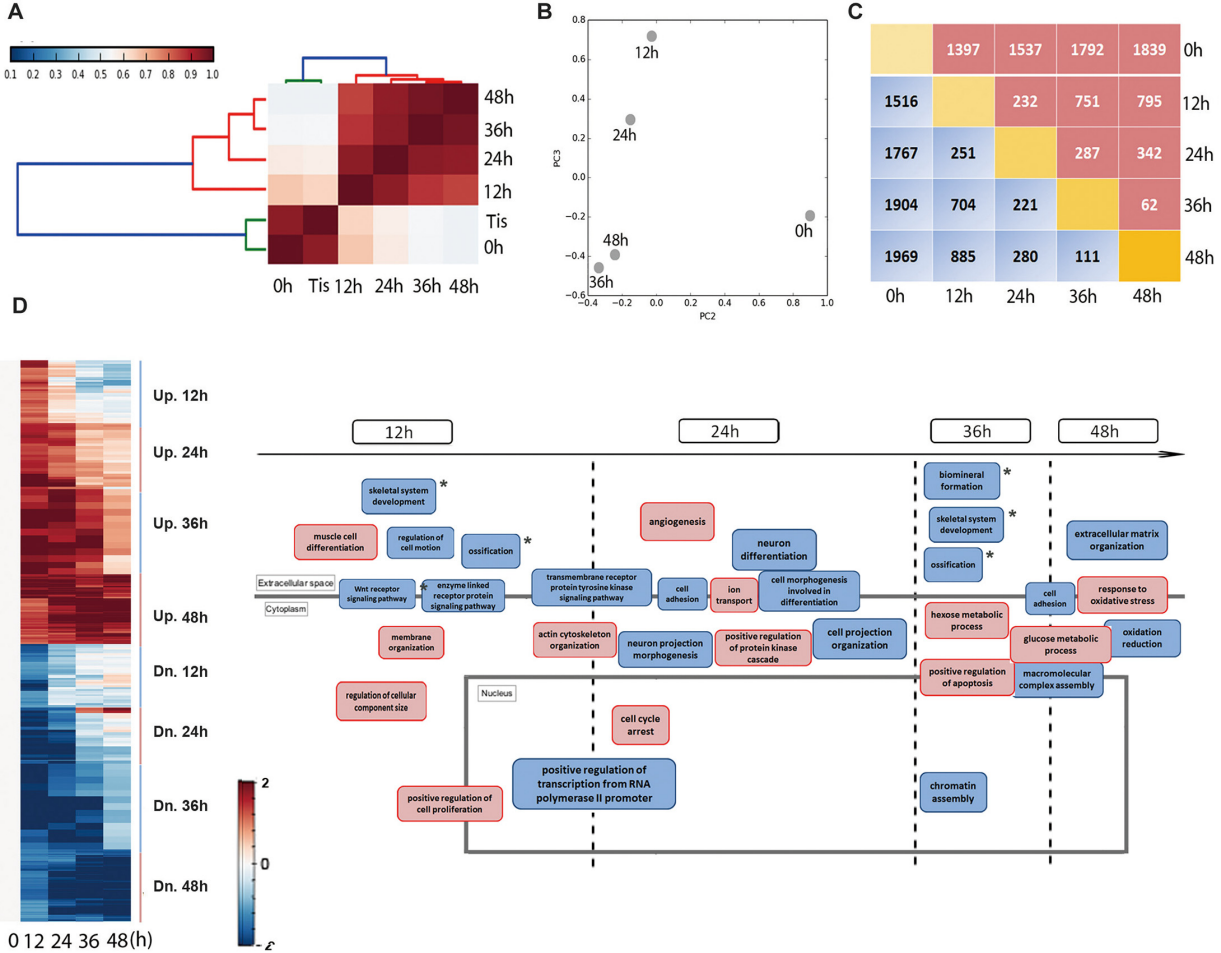
Global transcriptome profiles of mDMCs. **(A)** Hierarchical clustering analysis of dental mesenchymal tissue or cell samples. Tis, dental mesenchymal tissues. **(B)** Principal components analysis (PCA) showed the distances between various cell populations. Variation between two adjacent cell populations was reduced with time. **(C)** The number of differentially expressed genes between every two cell populations was listed. The red bracket indicates the number of upregulated genes and the blue indicates the downregulated genes. **(D)** Differentially expressed genes were divided into eight clusters according to their temporal patterns. Functional annotation analysis of genes in each cluster was performed, and the biological processes were reconstructed. The red bracket indicates processes involving the upregulated genes in the given time interval and the blue bracket indicates those involving the downregulated genes. * indicated the biological processes associated with tooth development.

To reveal the underlying mechanism of the impairment of odontogenic potential, we generated gene expression profiles from mDMCs in culture using RNA-seq. Correlation analysis performed at the cell population level showed that there were 2 main clusters (Fig 3A). Cluster I comprised freshly isolated mDMCs and dental mesenchymal tissues, indicating comparable odontogenic potential between the two components. Cell populations in cluster II exhibited a high mutual positive correlation and were those associated with cultured cells. However, the link between cluster I and mDMCs cultured for 12 h or 24 h was relatively strong compared with that of cells cultured for 36 h or 48 h. PCA was used to map cell populations, and a dominant component (PC3) matching the sequence of progressive loss of odontogenic potential was identified (Fig 3B). In addition, the magnitude of transcriptional changes during culture was reflected by the number of transcripts induced or repressed at a given time point. A remarkable number of genes were differentially expressed between freshly isolated and cultured mDMCs. However, culturing mDMCs for >24 h had a comparatively minor effect on transcription (Fig 3C).

To group the transcripts with similar behavior during culture, genes differentially expressed between freshly isolated and cultured mDMCs were divided further into eight clusters according to their expression patterns. Each cluster included genes exclusively upregulated or downregulated during the interval in culture (Fig 3D). Functional annotation analysis was performed to gain insight into the function of genes in each cluster. Much of the transcriptional change affected genes controlling proliferation and various ‘housekeeping’ activities such as transcription, cell motion, cell adhesion, and cytoskeletal organization. Interestingly, genes encoding skeleton- and ossification-related categories underwent downregulation during the intervals 0–12 h and 24–36 h, when the odontogenic potential of mDMCs evidently was degenerating. Throughout the culture duration, expression of genes controlling the negative regulation of cell proliferation, positive regulation of apoptosis, and cell-cycle arrest were enhanced. Collectively, mDMCs underwent a major perturbation in their biological processes that coincided with the loss of odontogenic potential.

### Phenotype alterations in cultured mouse dental mesenchymal cells

**Fig 4:**
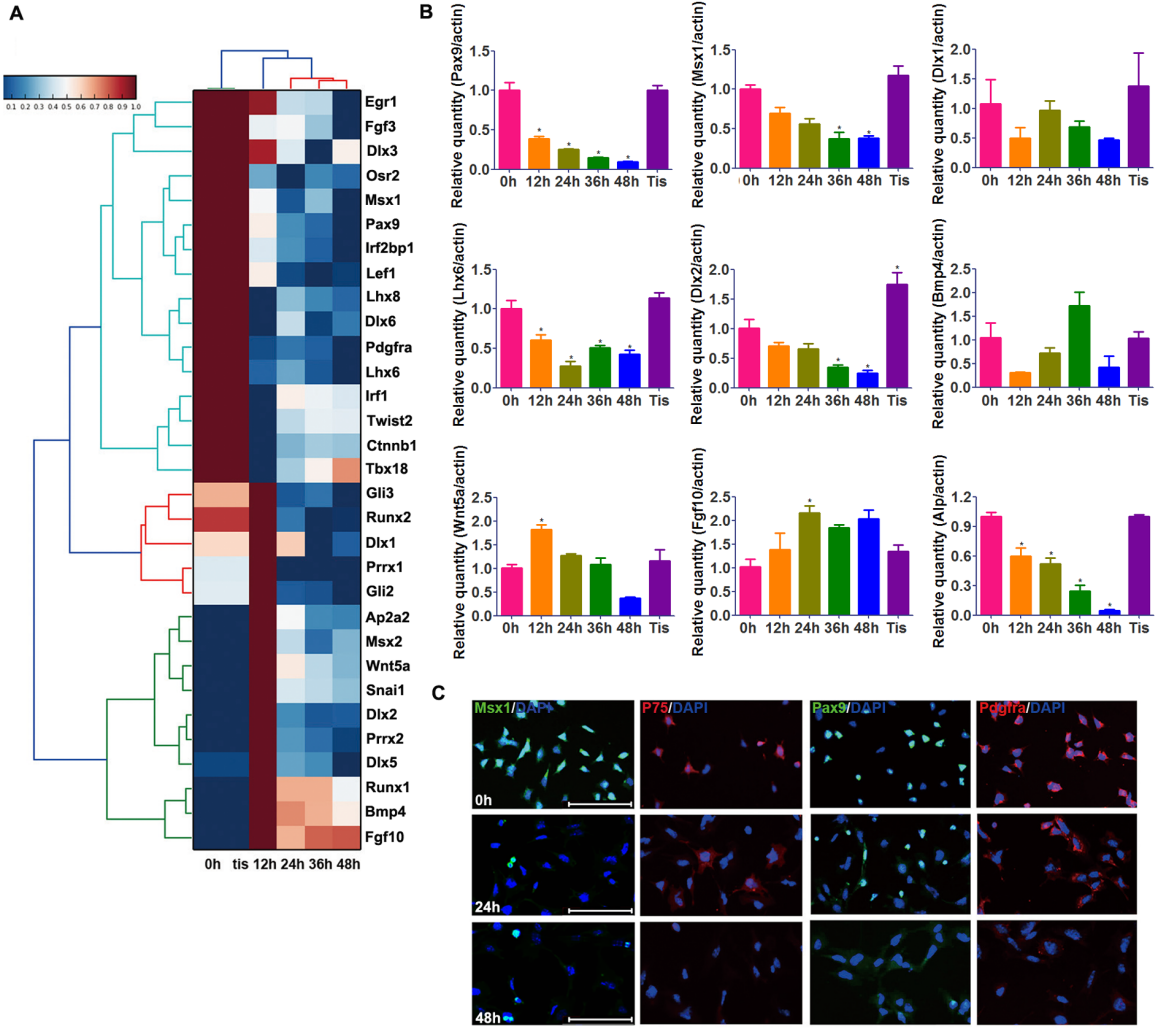
Phenotypic alteration of cultured mDMCs. **(A)** Heat map showed the decreased expression of dental mesenchyme-related genes in cultured mDMCs. **(B)** The mRNA levels of some dental mesenchyme-related genes. **(C)** Decreased expression of Msx1, Pax9, P75, and Pdgfrα in cultured mDMCs was examined by immunofluorescence assays. *p < 0.05 compared with the D0 cells. Scale bar: 100 μm.

The expression pattern of some dental mesenchyme-specific genes (*Msx1*, *Pax9*, *Lhx6*, *Dlx1*, and *Dlx2*) was examined using qRT-PCR and immunofluorescence analysis. In agreement with RNA-seq results (Fig 4A), these genes were downregulated in a temporal pattern approximately concordant with the loss of odontogenic potential (Fig 4B). Freshly isolated mDMCs were stained positive for Pax9 (~60%) and Msx1 (~73%). However, the fraction of cells positive for Pax9 (~25%) and Msx1 (~11%) declined remarkably during culture and was hardly detectable at 48 h (~1.5%; Fig 4C). mDMCs contained only a small fraction of cells positive for p75 (Ngfr, ~30%), which had almost completely disappeared at 48 h. In addition, expression of Pdgfrα was also decreased under *in vitro* conditions. However, the expression of *Bmp4*, *Wnt5a* and *fgf10* did not show a trend that is concordant with the loss of odontogenic potential (Fig 4B). Thus, the temporal pattern of some dental mesenchyme-specific genes like *Msx1* and *Pax9* corresponds to the change in odontogenic potential, and the decreased expression of these genes may contribute to the loss of odontogenic potential.

### Maintenance of odontogenic potential in mouse dental mesenchymal cells in culture

**Fig 5:**
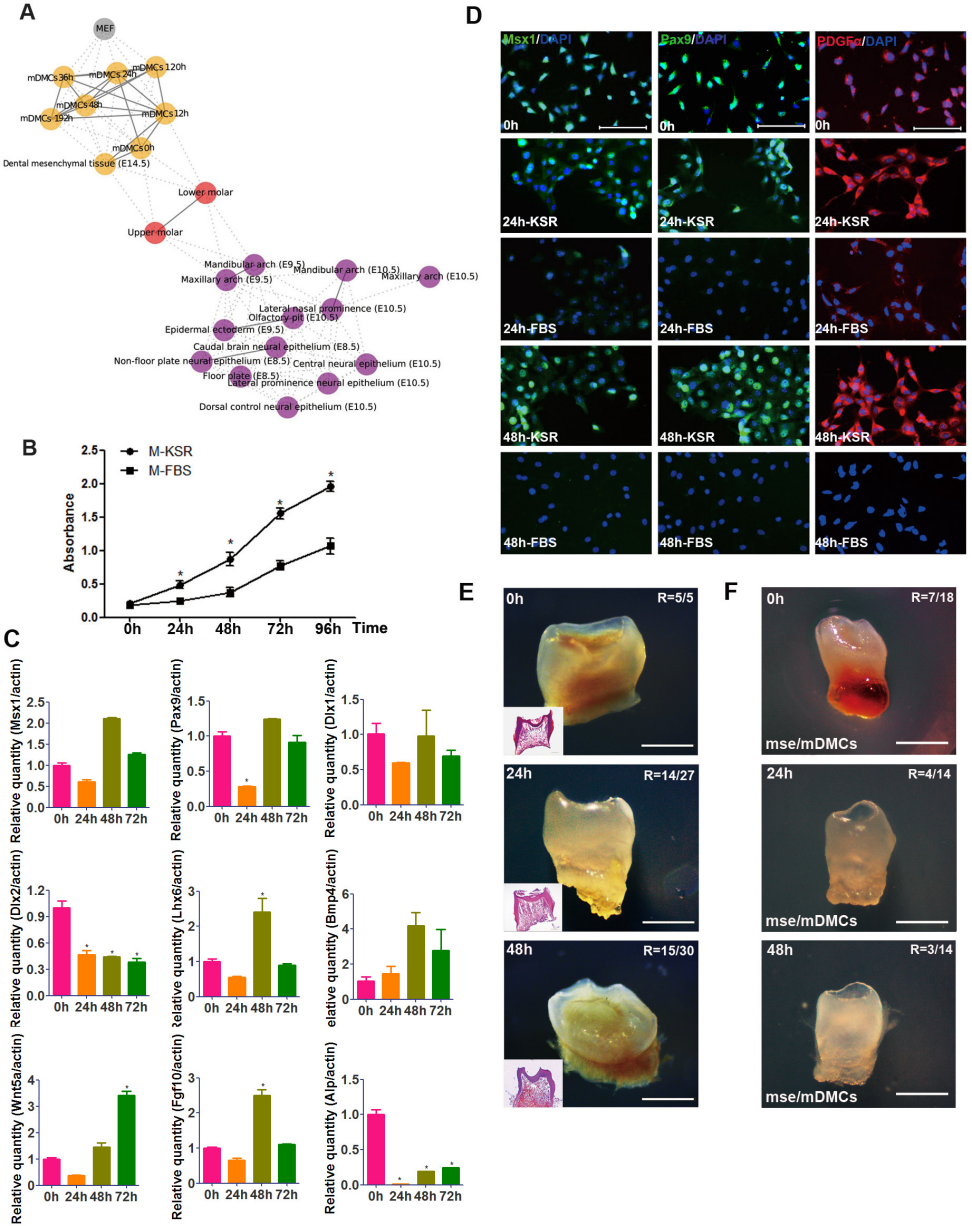
KSR, Fgf2 and Egf facilitate the maintenance of odontogenic potential in cultured mDMCs. **(A)** The relational network was constructed based on the strength of correlation between pairs of samples, showing the relationship of mDMCs to normal neural crest cell types. **(B)** mDMCs proliferated at a significantly higher rate in KSR-supplemented medium (M-KSR) than FBS-supplemented medium (M-FBS). **(C)** The mRNA levels of some dental mesenchyme-related genes in mDMCs cultured in medium with KSR. **(D)** Immunofluorescence analysis of Msx1, Pax9, and Pdgfrα in mDMCs cultured in medium with KSR or FBS. **(E)** Recombinants with mDMCs cultured for 24 h and 48h in KSR-supplemented medium developed into teeth after subrenal culture for 3 weeks. R, ratio of tooth formation. **(F)** Recombinants of epithelium from E10.5 mouse second-arch (mse) and cultured mDMCs developed into tooth-like structures. *p < 0.05 compared with the freshly isolated cells. Scale bar: D = 100 μm; E, F = 500 μm.

Since dental mesenchyme is derived from the neural crest, the RNA-seq data of tooth and neural crest obtained by others was then integrated with our data to understand the relationship of mDMCs to normal neural crest cell types. A relational network was built based on the strength of the correlation between pairs of samples (Fig 5A). Freshly isolated mDMCs were associated closely with the neural crest cells, but particularly with the upper and lower molars. When mDMCs were cultured, they came to resemble fetal fibroblasts more closely as they lost their odontogenic potential. We then investigated whether supplementation with growth factors essential for the development of neural crest and tooth could prolong the odontogenic potential. Egf and Fgf2 were incorporated into the culture medium to inhibit cell apoptosis and promote cell proliferation. Moreover, KSR was used to skip undefined factors in FBS and avoid cell differentiation towards fibroblasts. mDMCs in the new medium proliferated robustly, with a significantly higher rate than those in the FBS-supplemented medium (Fig 5B). The KSR-supplemented medium increased the mRNA expression of *Lhx6* and *Fgf10*, and maintained the expression of *Pax9*, *Msx1*, *Wnt5a*, *Bmp4*, and *Dlx1*, but failed to maintain the expression of *Dlx2* and *Alp* after 48 h in culture (Fig 5C). In addition, protein levels of Msx1, Pax9, and Pdgfrα in mDMCs cultured in KSR-supplemented medium were comparable to freshly isolated mDMCs but significantly higher than those in FBS-supplemented medium (Fig 5D). The protein level of Bmp4 was slightly increased in KSR-supplemented medium and slightly decreased in FBS-supplemented medium (S1 Fig). Surprisingly, the KSR-supplemented medium was capable of extending odontogenic potential of mDMCs, and tooth formation could be derived at 48 h (Fig 5E; 15/30; Table 2). The potential is lost gradually thereafter, and recombinants with mDMCs cultured for 72 h developed into only thin and tiny tooth-like structures with a ratio of 7/19. Since E14.5 dental mesenchyme also possesses the capability to instruct tooth formation when recombined with epithelia of non-dental origin, mDMCs cultured in KSR-supplemented medium were recombined with E10.5 mouse second-arch epithelium. Tooth-like structures were found in the recombinants of cultured mDMCs and second-arch epithelium (Fig 5F, Table 2), confirming the retained odontogenic potential in cultured mDMCs. Thus, it is possible to maintain the odontogenic potential of mDMCs *in vitro* by modulating the composition of culture medium.

### The effects of KSR, Fgf2, and Egf on cultured mDMCs

**Fig 6:**
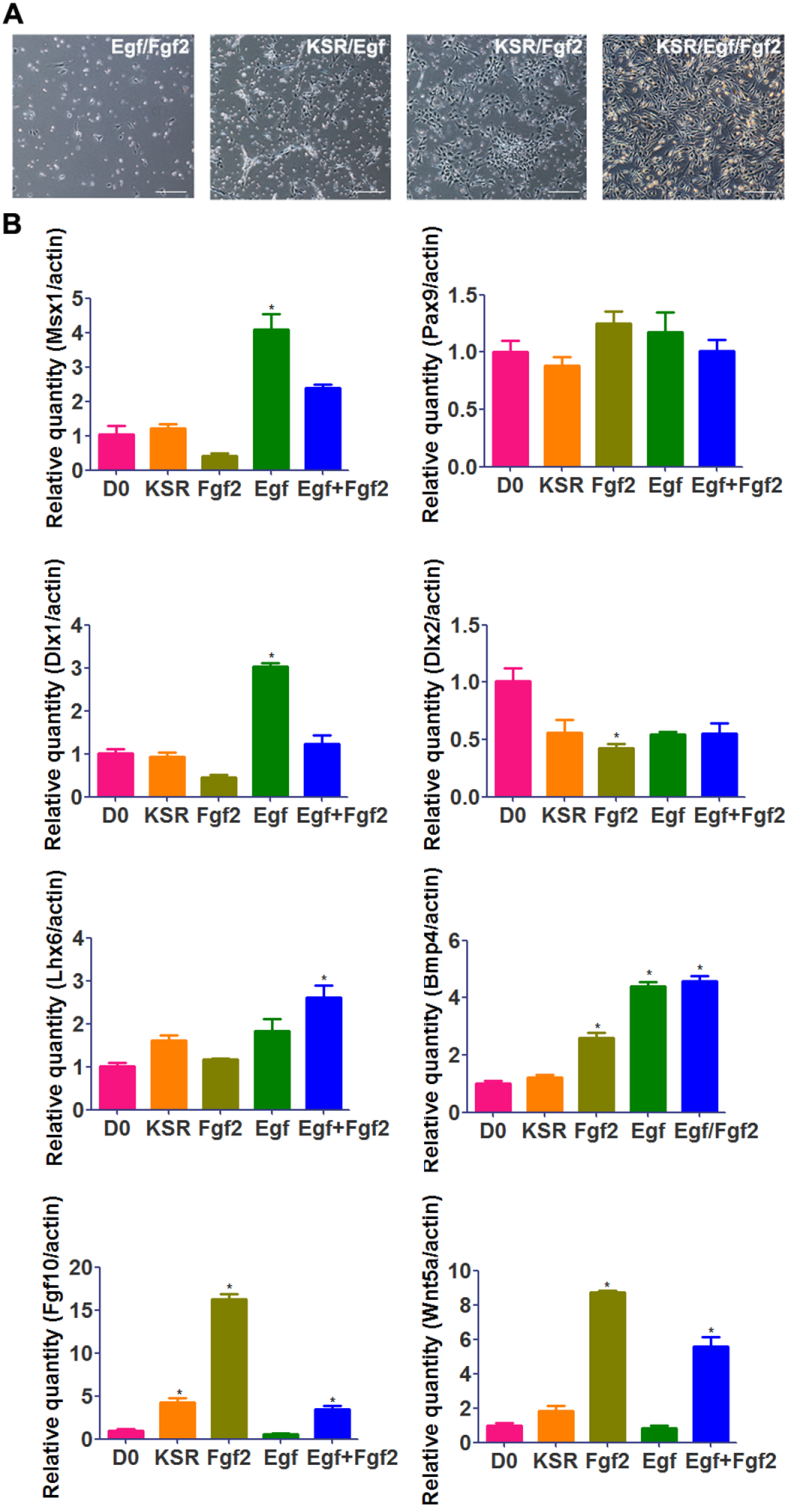
The effects of KSR, Fgf2, and Egf on cultured mDMCs. **(A)** Images of cells cultured in the medium with Egf/Fgf2, KSR/Egf, KSR/Fgf2, and KSR/Egf/Fgf2. Scale bar: 50 μm. (B) The mRNA levels of some dental mesenchyme-related genes in freshly isolated (D0) mDMCs and mDMCs cultured in KSR-, KSR/Fgf2-, KSR/Egf-, and KSR/Egf/Fgf2-supplemented medium. *p < 0.05 compared with the D0 cells.

To identify the differential effect of individual supplement, one or two supplements were used to cultured mDMCs. When Egf/Fgf2 were used and KSR was removed from the medium, mDMCs poorly attached to the culture flasks. When Egf or Fgf2 was removed, the cells displayed normal morphology but proliferated relatively slowly (Fig 6A). KSR/Fgf2 increased the expression of *Fgf10*, *Wnt5a*, and *Bmp4*, but decreased the expression of *Dlx2*. KSR/Egf increased the expression of *Msx1*, *Dlx1*, and *Bmp4*, while KSR increased the expression of *Fgf10* (Fig 6B). Moreover, the downstream effects of Egf and Fgf2 were predicted using bioinformatic approaches. Egf was predicted to restore the expression of *Egr1*, *Egr2*, *Jund*, and *Fos* (S2 Fig). Fgf2 was predicted to restore the expression of *Runx2* and *Twist2* (S3 Fig). Thus, KSR is essential for the survival of mDMCs, and both Egf and Fgf2 induced the expression of some dental mesenchyme-related genes.

## Discussion

mDMCs possess rigorous odontogenic potential; nevertheless, *in vitro* expansion of odontogenic mDMCs has proven to be a great challenge. In the present study, cultured mDMCs rapidly lost their odontogenic potential. The loss of epithelial-mesenchymal interaction and cell-cell communication may account for the loss of odontogenic potential when cells are grown as a monolayer. A previous study demonstrated that the odontogenic potential of mDMCs was lost rapidly in the course of culture and mDMCs cultured for 24 h did not support tooth formation [20]. However, we successfully maintained the odontogenic potential of mDMCs for 48 h in a KSR-supplemented medium, suggesting a new route to long-term maintenance of odontogenic potential.

Tooth development involves epithelial-mesenchymal interactions mediated by conserved signals in the signal families BMP, FGF, Hh, and Wnt [1–2]. Odontogenic potential shifts to dental mesenchyme at the bud stage [4–5]. FGF and BMP are potential signals transmitting odontogenic potential from the epithelial to mesenchymal compartment [30–31]. Unexpectedly, the temporal pattern of *Bmp4* and *Fgf10* did not correspond to the change in odontogenic potential in our study. Expression of *Fgf3* decreased dramatically in cultured mDMCs. However, restored expression of *Fgf3* does not rescue the odontogenic potential of cultured mDMCs [20]. Hence, other genes could also contribute to the loss of odontogenic potential. BMPs and FGFs induce expression of several mesenchymal transcription factors, including *Msx1*, *Msx2*, *Pax9*, *Dlx1*, *Dlx2*, *Lef1*, and *Lhx6* [32–33]. Several of these genes perform essential functions during tooth development, because deletion of their functions in transgenic mice results in arrest in tooth development [34–37]. In our study, *Msx1*, *Pax9*, *Dlx1*, *Lef1*, and *Lhx6* were significantly reduced in cultured mDMCs, leading to impairment of the odontogenic potential and, ultimately, the failure of tooth formation. Although Bmp4 was shown to activate the expression of Msx1 [38], Bmp4 was maintained and Msx1 was downregulated in FBS-supplemented medium. The deregulation of other growth factors like Fgf8 [38] and transcription factors like Crebbp and Sp1 may lead to the decreased expression of Msx1 [39]. Recently, transfection of *Pax9* and *Bmp4* into iPS-derived NCLCs has been shown to promote differentiation into odontoblast-like cells [40]. Our results indicated that overexpression of genes other that *Pax9* and *Bmp4* are needed to confer odontogenic potential on iPS-derived NCLCs.

Although the molecular mechanisms regulating the odontogenic potential are not fully understood, our data demonstrated that medium supplemented with KSR, Fgf2, and Egf prolonged the odontogenic potential of mDMCs. KSR is a defined and serum-free formulation used to support the growth of stem cells since it prevents spontaneous differentiation. Transferrin, which is contained in KSR, is necessary for development of cap-staged tooth in organ culture and stimulates cell proliferation in tooth germs [41]. In addition, insulin in KSR facilitates the proliferation of stem cells. In the present study, KSR is essential for the survival of mDMCs and cells proliferated robustly in KSR-supplemented medium. Fgf2 and Egf were also employed to maintain the odontogenic potential of mDMCs. Fgf2 facilitates the maintenance of the specific properties of various progenitor cell types, including bone marrow and dental pulp stem cells [42–43]. Egf induces the proliferation of dental tissue, and Egf down-regulation during early mouse development results in impaired tooth formation. We found that both Egf and Fgf2 contributed to the expression of certain genes that are important for tooth development. Coincidently, the combination of Egf and Fgf2 has been used to derive neural crest cells from embryonic stem cells [44–45]. Neural crest cells contribute to a variety of derivatives including teeth and are found mainly in the condensed dental mesenchyme under the enamel organ [46–47]. In the present study, the transcriptomic link between mDMCs and the neural crest was abrogated when mDMCs were cultured in FBS-supplemented medium, but Fgf2 and Egf which trigger mitosis in neural crest cells facilitated the maintenance of odontogenic potential.

mDMCs cultured in KSR-supplemented medium for 72 h also expressed the dental mesenchyme-specific markers, but the morphology of regenerated tooth-like structure was abnormal. Thus, other regulatory signals should be considered to maintain the odontogenic potential of mDMCs. Gene expression profiling was used to explore the possible mechanism in the present study. The relationships among the transcriptome profiles of mDMCs recapitulated the evolvement of odontogenic potential impairment. Genes controlling the skeleton- and ossification-related activities were downregulated, providing insights into the optimization of culture medium in the future. Besides, considering the cell heterogeneity in the dental mesenchyme [48], the inability to sustain a certain cell population may also lead to the culture-induced impairment of odontogenic potential and the altered transcriptomic profiles. However, further studies are still needed to identify the transcriptomic profiles for different cell populations in dental mesenchyme before we can determine the transition of cell heterogeneity. Moreover, three-dimensional (3D) culture scaffolds mimic the *in vivo* microenvironment that is essential to the function of cells. Thus, the 3D culture scaffolds, instead of traditional two-dimensional (2D) culture methods, would manage to prolong the properties of cultured mDMCs, and therefore exhibits promising potential in tooth regeneration.

In summary, our results showed the culture-induced impairment of odontogenic potential and provided a panoramic view of the transcriptomic landscape of cultured mDMCs. Notably, a new culture micromilieu ameliorated the impairment of odontogenic potential in 48 h, providing a clue for long-term culture of mDMCs without compromising their odontogenic potential.

## Supporting Information

S1 Fig Immunofluorescence analysis of Bmp4 in mDMCs cultured in FBS- or KSR-supplemented medium. Scale bar: 100 μm.
(TIF)

S2 Fig Downstream effects of Egf predicted by Ingenuity pathway analysis. (TIF)

S3 Fig Downstream effects of Fgf2 predicted by Ingenuity pathway analysis. (TIF)
