# Supplementary figures and images for: Remission for Loss of Odontogenic Potential in a New Micromilieu In Vitro
Source: PLoS One. 2016 Apr 6;11(4):e0152893. doi: 10.1371/journal.pone.0152893 (PMC4822848; doi:10.1371/journal.pone.0152893)

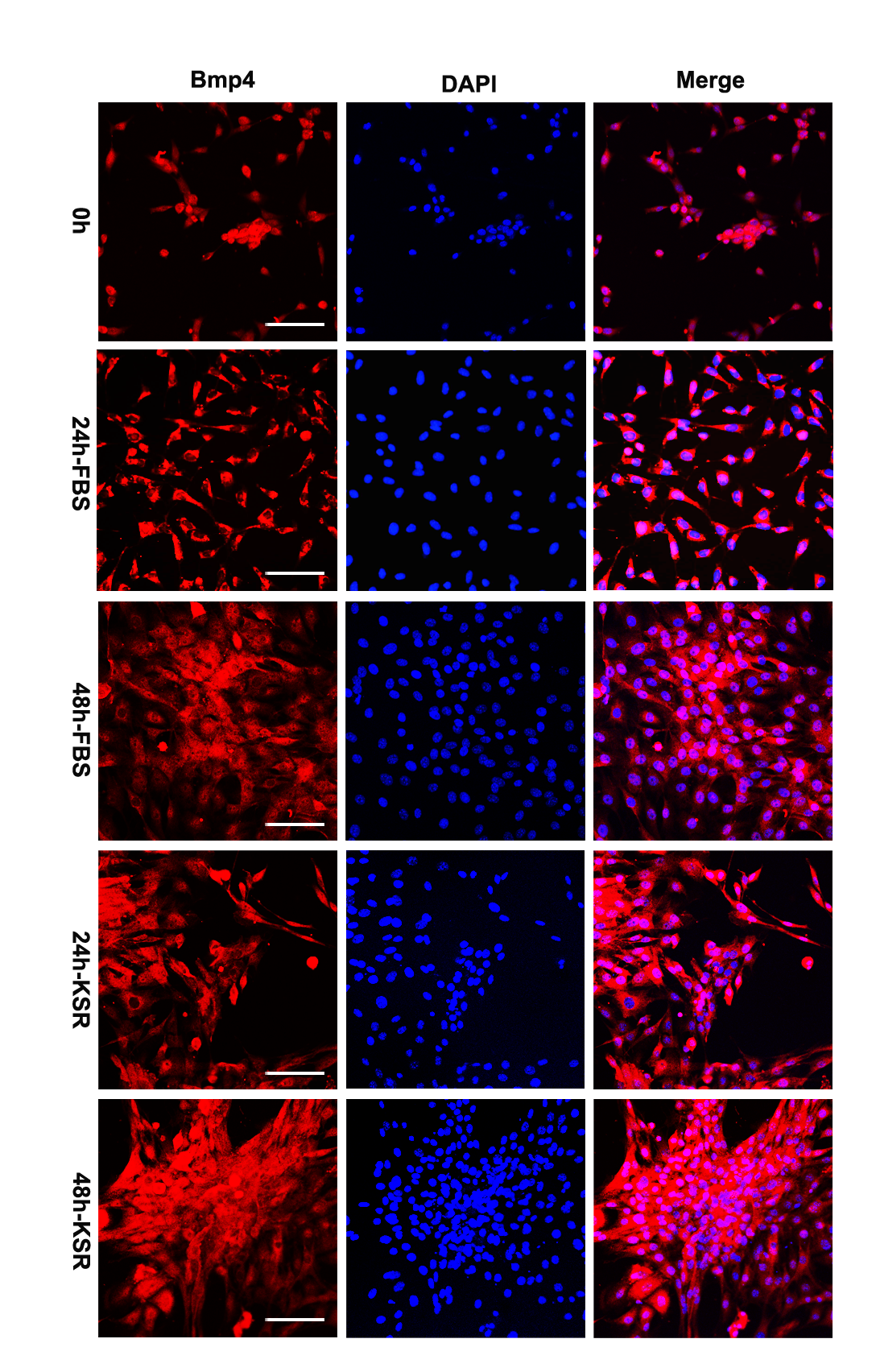

Supplement: S1 Fig — Scale bar: 100 μm. (TIF) [file pone.0152893.s001.tif]

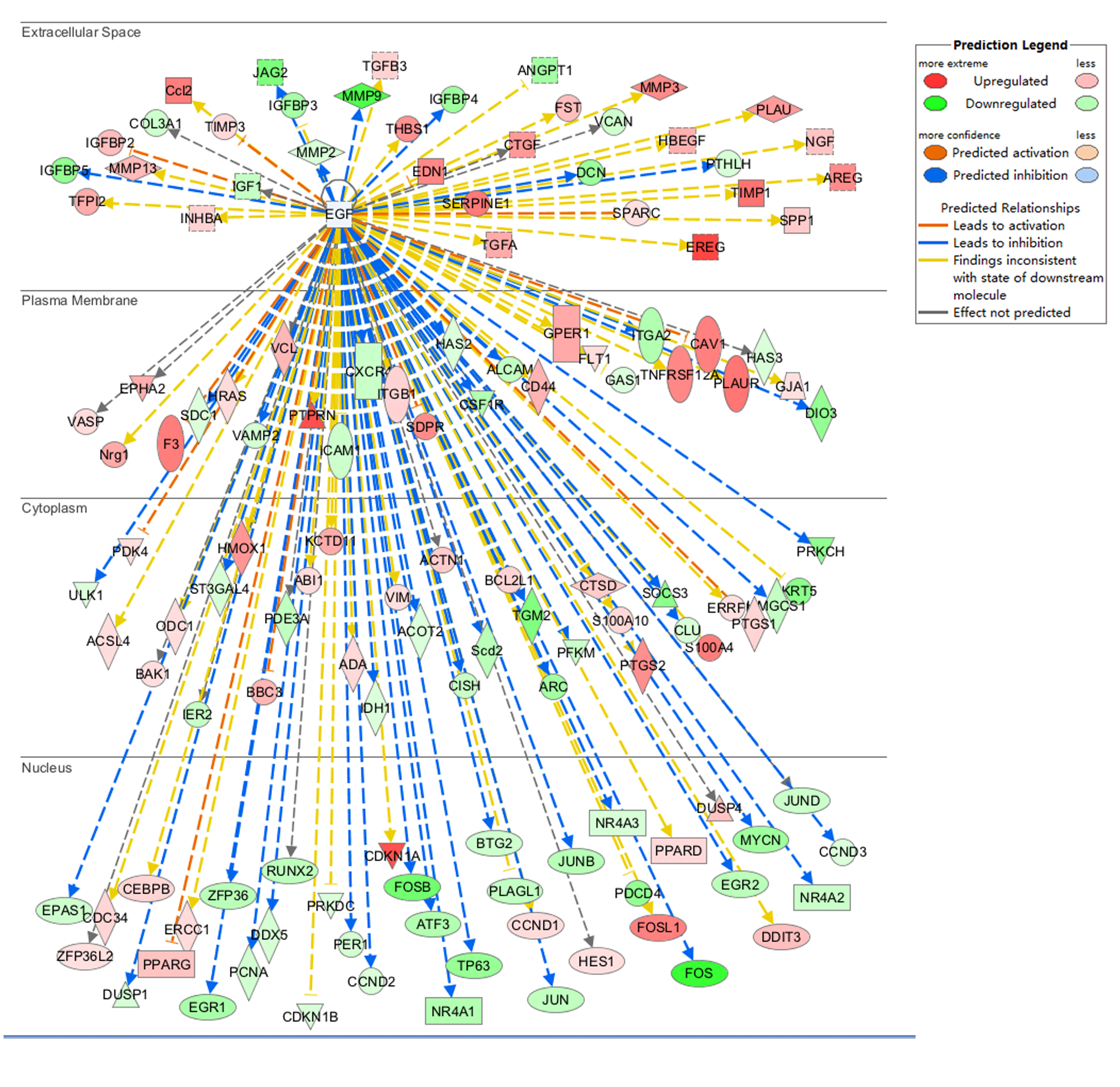

Supplement: S2 Fig — (TIF) [file pone.0152893.s002.tif]

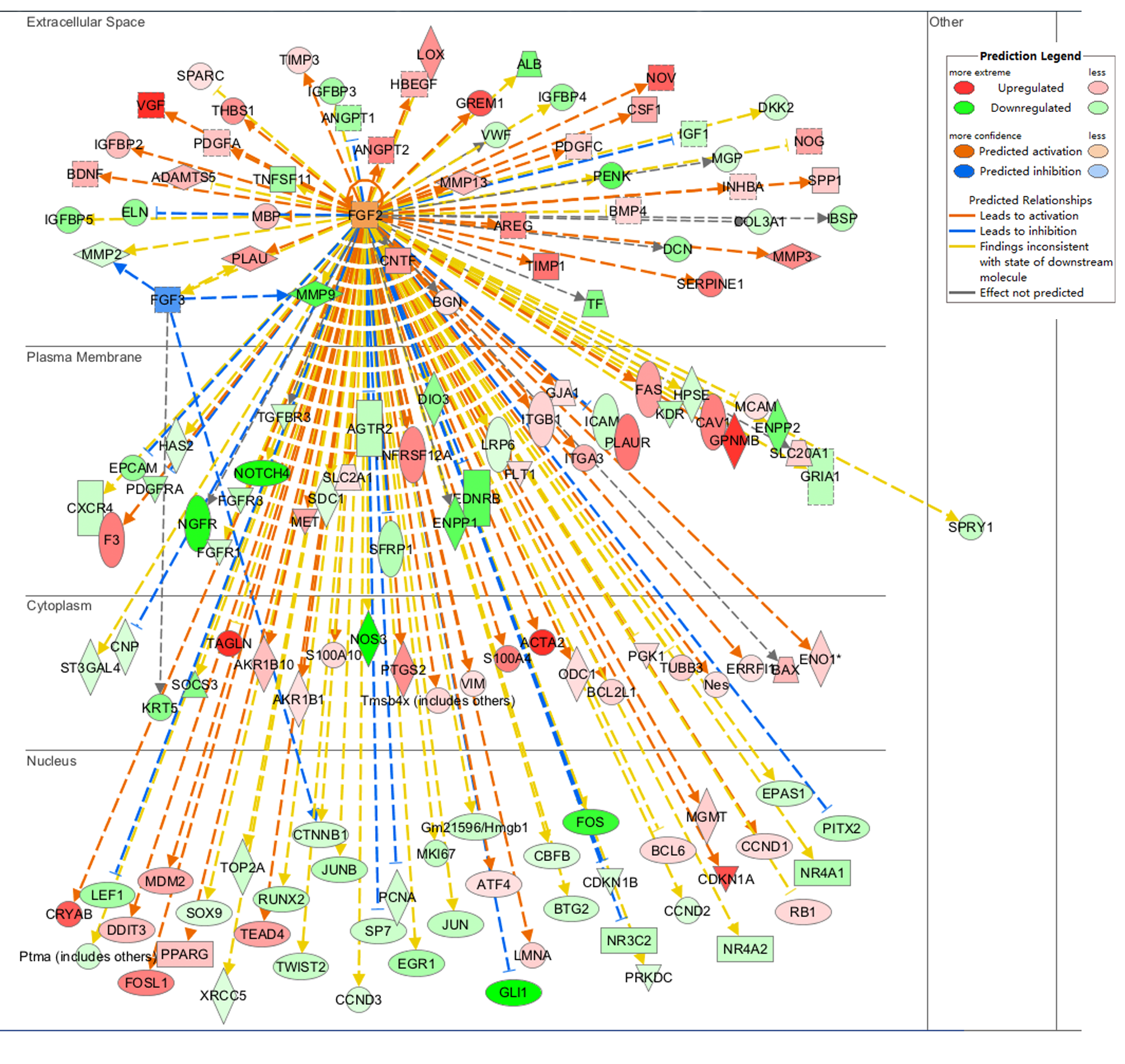

Supplement: S3 Fig — (TIF) [file pone.0152893.s003.tif]
